# New Drugs and Preparations

**Published:** 1894-07-14

**Authors:** 


### NEW DRUGS AND PREPARATIONS.

[We shall be glad to receive, at our Office, 428, Strand, London, W.C., from the manufacturers, specimens of all new preparations and drugs which may be brought out from time to time.]

### BORAX IN EPILEPSY.

Dr. W. Alexander (*Liverpool Med.-Chir. Journ.*, July, 1893) contributes an article on the treatment of epilepsy in which his experience of borax is given at some length. Borax alone proved to be of little value in most cases, but along with small doses of bromide of potassium, or, better still, bromide of sodium, it had a more satisfactory effect. He gave the following prescription usually: ℞ Sodii biborat., gr. 200; sodii bromid., gr. 50; syrup, ʒj.; aq. ad. ℥x. Sig., 1 ounce three or four times daily in water, after food. Not only the fits, but the mental condition of the patients were greatly benefited. There are, however, certain drawbacks to the use of the drug. 1. It may cause gastric disturbance, with pain, flatulence, and loss of appetite, but these symptoms may generally be obviated by giving it well diluted and after food. 2. Skin eruptions—erythema, and sometimes almost eczema—are occasionally observed after taking the medicine for some time. The itching and irritation may necessitate stoppage of the drug. It sometimes subsides even when the drug is persevered with, but alkaline baths or vaseline relieve it greatly. 3. Loss of hair occurs sometimes, but very rarely. In the case of a boy there was complete loss of hair over the whole body, which came on after the borax had been taken for some months; the same occurred in another boy and in two adults, while localised alopecia has often been observed. The reason of the alopecia is not known, but the hair has always grown in again. 4. Loss of weight and emaciation seem to occur along with the administration of borax. Mairet (*Lancet*, May 6th) finds that borax produces toxic effects—gastric disturbances, œdema of legs, loss of hair, and conjunctivitis—in nearly all the cases in which it is given. He recommends small doses, from 8 to 15 grains daily, gradually and carefully increased to 60 or 70 grains. If the attacks are not lessened by 120 grains daily, no benefit can be expected from it. He does not consider that the advantages of the remedy compensate for its great disadvantages.

### SOLUBILITY OF SALTS OF QUININE AND THEIR HYPODERMIC USE.

*L'Union Médicale* (1893) gives the solubilities of salts of quinine in water as follows, with reference to their hypodermic use in the treatment of severe malarial poisoning: 1 part of neutral hydrochlorate in ·66 of water; 1 neutral sulphovinate in ·70; 1 neutral lactate in 2·00; 1 basic sulphovinate in 3·30; 1 neutral hydrobromate in 6·33; 1 neutral sulphate in 9·00; 1 basic lactate in 10·29; 1 basic hydrochlorate in 21·40; 1 basic hydrobromate in 45·02; and 1 basic sulphate in 581. The neutral hydrobromate is therefore the most suitable for hypodermic use, as it is most soluble and contains a large quantity of the alkaloid. The addition of antipyrin is found to aid the solubility. Briz has used a new salt, the hydrochloro-sulphate of quinine—a combination of hydrochloric and sulphuric acid with the alkaloid—soluble 1 in 1 of water. It is rapidly absorbed, produces no irritation, and particularly in children, who object to take quinine by the mouth, the results have been admirable. (*B. M. J.*, Ep., Mar. 24, 1894). Corre (*Bull. Gén. de Thérap.*, Oct. 1892) points out that all quinine salts are capable of producing methæmoglobin, with consequent methæmoglobinura and possible biliary poisoning. Such an action must be very rare, although it has been repeatedly reported with the other antipyretics antifebrine and antipyrin.

### SALACETOL.

This is a substitute for salol proposed by Professor Bourget (*Correspondenzbl. f. Schweizer Aerzte*, No. 14, 1893), salol being sometimes dangerous on account of the large amount of phenol set free from it in the bowel. It is a compound of acetol and salicylic acid having the composition $C_6 H_4$ OH COO $CH_2$ CO $CH_3$ and containing 75 per cent. of salicylic acid. It is a white cystalline powder, insoluble in water. When it reaches the alkaline duodenum it gradually breaks up into salicylic acid and acetol, the latter being quickly eliminated as acetone, while the former acts as an intestinal antiseptic, antirheumatic, &c. If given with castor oil it is more rapidly decomposed and is more efficient, probably because the increased intestinal secretion acts more powerfully on it. Bourget gives it in 30 to 45 grain doses along with castor oil, taken in the morning on an empty stomach, and this may be repeated two or three mornings in succession. Children may take seven grains safely. Bourget has found it effective in summer diarrhœa, cholera nostras, ordinary diarrhœa, and for disinfecting the genito-urinary passages, as well as in chronic and subacute rheumatism.

